# Fractionated irradiation induced radio-resistant esophageal cancer EC109 cells seem to be more sensitive to chemotherapeutic drugs

**DOI:** 10.1186/1756-9966-28-68

**Published:** 2009-05-27

**Authors:** Li Xie, Xianrang Song, Jinming Yu, Ling Wei, Bao Song, Xingwu Wang, Liyan Lv

**Affiliations:** 1 Cancer Research Center, Shandong Cancer Hospital, 440 Ji-Yan Road, Jinan, Shandong Province 250117, PR China

## Abstract

**Background:**

Chemo-radiotherapy, a combination of chemotherapy and radiotherapy, is the most frequent treatment for patients with esophageal cancer. In the process of radiotherapy, the radiosensitive cancer will become a radio-resistant one.

**Methods:**

In order to detect the chemotherapeutic drug sensitivity in radio-resistant cancer cells and improve the therapy efficiency, we firstly established a radio-resistant esophageal cancer cell model (referred to as EC109/R) from the human esophageal squamous cell carcinoma cell line EC109 through fractionated irradiation using X-rays. The radio-sensitivity of EC109/R cells was measured by clonogenic assay. To detect the drug sensitivity for EC109/R compared to its parent cells, we employed MTT method to screen the effectiveness of five different drugs commonly used in clinical therapy. The ratio of apoptosis was examined by flow cytometry.

**Results:**

EC109/R cells were more sensitive to 5-fluorouracil, doxorubicin, paclitaxel and etoposide, but tolerant to cisplatin compared to its original cells.

**Conclusion:**

Our study implies that fractionated irradiation induced radio-resistant esophageal cancer cell is more sensitive to certain kind of chemotherapeutic drugs. It provides evidence for choosing the sequence of radiotherapy and chemotherapy in esophageal cancer.

## Background

Esophageal squamous cell carcinoma (ESCC) has commanded increased attention in the past three decades because of changing epidemiologic patterns and expanded treatment options [1-4]. Worldwide, esophageal cancer is the sixth leading cause of cancer death, and its 5-year survival rate in the United States is 14.9%, being responsible for 4% of all cancer deaths annually. The age-standardized incidence rate in China was the highest in the world. Surgical treatment is the mainly way for localised esophageal carcinoma (stage I-III), but is very limited effective for stage III [5]. Patients undergoing surgery alone had a median survival ranging from 13 to 19 months and a 5-year survival rate of 15% to 24%.

The introduction of adjuvant chemo- and radiotherapy has improved the prognosis of patients with ESCCs, particularly those with high potential for lymph node metastasis [6,7]. Radiotherapy in particular has played a key role in the control of tumor growth in esophageal cancer patients. This mode of therapy is considered to improve resection rates, increase survival time, and decrease lymph metastases. However, the 5-year survival rate with conventional doses of radiation alone is 0% to 10% [8]. One of the reasons for this low survival rate is the insensitivity of esophageal cancer to radiotherapy, which decreases the ability to cure or delay progression of disease in these patients. Recently, chemo-radiotherapy, a combination of chemotherapy and radiotherapy, is the most frequent treatment for patients with esophageal cancer [9-12], and a complete histopathological response is achieved in 20%–40% of cases. This combination therapy has significantly improved median survival and reduced late relapses in patients with ESCCs. Therefore, suitable chemotherapy agents for esophageal cancer, especially for radio-resistant esophageal cancer are urgently needed.

The purpose of our experiment is to detect the chemotherapeutic drug sensitivity in radio-resistant cancer cells and improve the therapy efficiency. In the present study, we first established a radio-resistant cell model EC109/R from the human ESCC cell line EC109, by fractionated irradiation using X-rays. Then the efficiency of chemotherapeutic drug, cisplatin, 5-fluorouracil, doxorubicin, paclitaxel, or etoposide, was screened in EC109 and EC109/R cells.

## Methods

### Cell line and cell culture

EC109 cells, a well differentiated human ESCC cell line, were provided by Cancer Institute and Hospital, Chinese Academy of Medical Sciences. Cells were maintained in Dulbecco's modified Eagle's medium (DMEM, GIBCO, USA) containing 10% heat-inactivated fetal bovine serum (FBS, GIBCO), 100 U/ml penicillin, 100 U/ml streptomycin and 2 mM L-glutamine at 37°C in a humidified atmosphere of 5% CO_2_. Cells were passaged every 2–3 days to maintain exponential growth.

### Chemotherapeutic Agents

Cisplatin, 5-fluorouracil, doxorubicin, paclitaxel and etoposide were of analytical grade and were purchased from Sigma-Aldrich. They were dissolved in normal saline at various concentrations. Drug treatment involved continuous exposure to the compounds.

### Establishment of radio-resistant cell line

The method for establishing radio-resistant cell line by fractionated irradiation has been described previously[13]. Briefly, the cell line was first grown to approximately 60% confluence in 25-cm^2 ^culture flasks. Cells were irradiated with 10 Gy of X-ray irradiation, from a linear accelerator (6-MV X-ray), at a rate of 3 Gy/min. One cm thick of tissue-equivalent bolus was placed on top of the plate to ensure homogeneity. And then cells were returned to the incubator. When they reached approximately 60% confluence, the cells were again irradiated with 10 Gy of X-ray. The fractionated irradiations were continued until the total concentration reached 80 Gy. The radio-resistant cell subline was then established. The parental cells were subjected to identical trypsinization, replating, and culture conditions, but were not irradiated. For all assays on irradiated cells, there was at least a four-week interval between the last 10 Gy fractionated irradiation and the experiment.

### Assay for radiosensitivity

Cell survival after X-ray irradiation was measured by clonogenic assay. Cells were plated in six-well culture plates, and were irradiated at different concentration ranging from 0 to 12 Gy. The appropriate plating density was aimed to produce 20–100 surviving colonies in each well. These cells were incubated at 37°C for 10–14 days (three wells in each radiation concentration). After fixation with acetic acid-methanol (1:4) and staining with diluted crystal violet (1:30), colonies consisting of 50 cells or more were counted under a light microscope. The triplicate colonies were averaged and divided by initial seeded cells to yield survival rate of clones for each concentration, and the surviving fraction was determined. All survival curves represent at least three independent experiments.

### Detection of apoptotic cells

Apoptosis was evaluated using the Annexin V-FITC Apoptosis Detection Kit (BD Biosciences Pharmingen, San Jose, CA, USA) followed by FACS analysis. Cells were treated with trypsin-EDTA in PBS at pH 7.5, washed with normal medium and cold PBS, and then resuspended in 1× binding buffer. Five μl of annexin V and ten μl of propidium iodide were added to the cells, vortexed, and incubated for 15 minutes in the dark. Finally, 400 μl of 1× binding buffer was added, and samples were evaluated by flow cytometry.

### MTT cell viability assay

Drug-induced cytotoxicity was evaluated by conventional MTT cell viability assay as previously reported [14,15]. Briefly, 1 × 10^4^/well EC109 or EC109/R cells were seeded in 96-well plates and cultured in DMEM media supplemented with 10% FBS for 8 h. They were exposed to various concentrations of cisplatin (3.33–63.3 μM), 5-fluorouracil (0.07–4.93 mM), doxorubicin (0.53–7.36 μM), paclitaxel (3.12–100 nM) or etoposide (1–16 μM) for 48 h in a CO_2 _incubator. Ten μl of 5 g/L 3-(4,5-dimethylthiazol-2-yl)-2,5-diphenyltetrazolium bromide (MTT) solution was added to each well for 4 h at 37°C. Subsequently the formazan crystals were solubilized with 100 μl of 10% sodium dodecyl sulfate (SDS) in 0.01 M HCl for 24 h. Absorbance at 570 nm relative to a reference wavelength of 630 nm was determined with a microplate reader (Bio-rad 680, Bio-rad, USA). The concentrations resulting in 50% inhibition of cell growth (IC_50 _values) were calculated.

### Statistical analysis

A statistical analysis was performed using two-tailed Student's *t*-test to assess the statistical significance of treated groups versus control groups. The results with *P*-values of less than 0.05 were considered to be statistically significant.

## Results

### Establishment of cell subline resistant to irradiation

**Figure 1:**
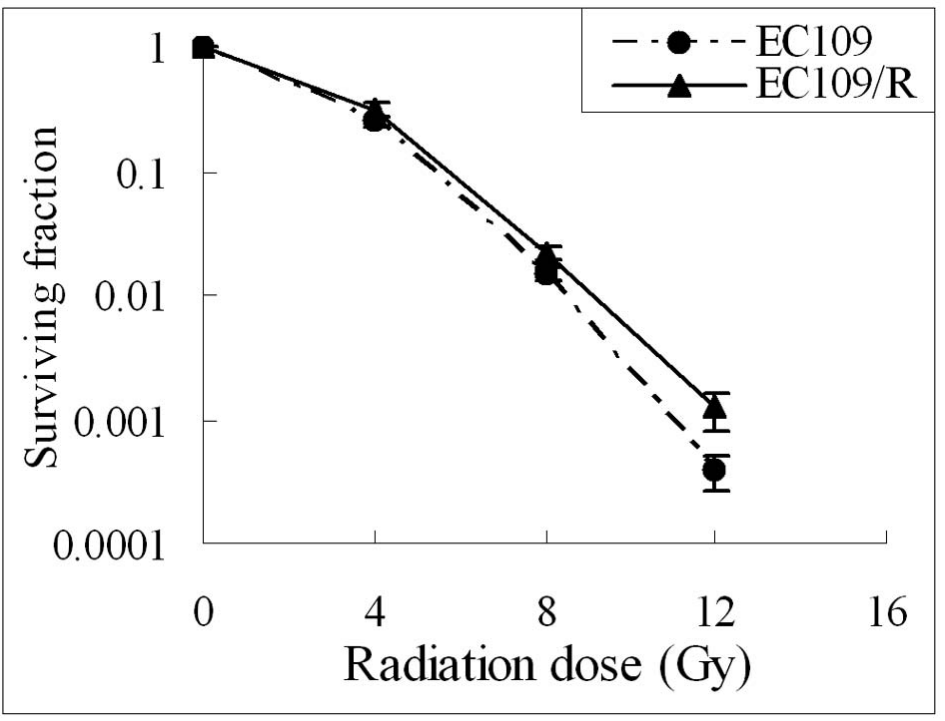
**Radiation cell survival curves for EC109 and EC109/R cells**. The colony formation assay was described in Materials and methods. Data represent means with standard deviation (SD) from three independent experiments. There was a significant difference in surviving fraction between parent and radio-resistant cells (p < 0.05).

The EC109 cells were treated repetitively with 10 Gy of X-ray irradiation, with about 20 days recovery allowed between each fraction until the total concentration reached 80 Gy. The radio-resistant cells were named EC109/R. The clonogenic assay was used to analyze their radiosensitivity after 0–12 Gy irradiation. Figure 1 shows the survival curves of parent and radio-resistant cells. Surviving fractions are shown in Table 1. The subline EC109/R was more radio-resistant to irradiation than the parental cell line EC109. Therefore, we considered the subline EC109/R as a radio-resistant cell line and the radio-resistant subline maintained a relative radio-resistant phenotype for at least two months after cessation of fractionated irradiation (data not shown). For the following assay on EC109/R cells, there was a six-week interval between the last 10 Gy fractionated irradiation and the experiment.

**Table 1:** Comparison of surviving fraction between EC109 and radio-resistant EC109/R cells exposed to various radiation concentration

Cell line	Radiation concentration		
	
	4 Gy	8 Gy	12 Gy
EC109	0.2545 ± 0.023	0.01493 ± 0.0018	0.00038 ± 0.00012
EC109/R	0.3197 ± 0.043	0.02209 ± 0.0033	0.00122 ± 0.0004

p-value	0.032522	0.035813	0.037994

Values reflect mean ± standard deviation (SD).

### Cell proliferation assay

**Figure 2:**
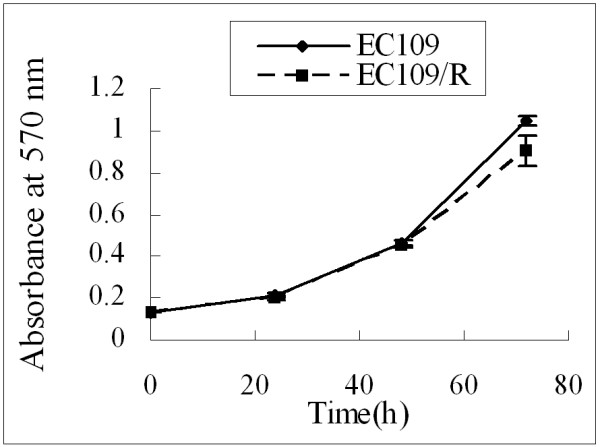
**Cell proliferation assay of EC109 and EC109/R cells**. Cells were cultured in 96-well plates for 0, 24, 48 and 72 h. Cell viability was determined by MTT assay. Each point represents the mean ± SD of triplicate experiments (p > 0.05).

To assess cell proliferation of EC109/R, cell viability was determined by MTT assay. Aliquots of 2 × 10^3^/well EC109 or EC109/R cells were cultured in 96-well plates for 0, 24, 48, and 72 h. The absorbance intensity of the MTT product was detected. As shown in Figure 2, there was no significant difference in cell growth after three repetitive treatments between EC109 and EC109/R (P > 0.05). Each point in figure 2 represents the mean ± SD of triplicate experiments.

### Irradiation-induced apoptosis in EC109/R cells

**Figure 3:**
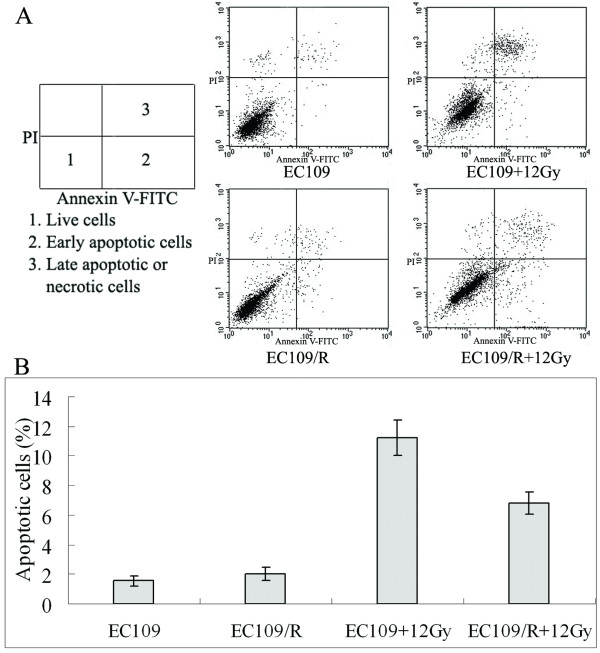
**Irradiation-induced apoptosis in EC109 and EC109/R cells**. Cells (1 × 10^6 ^each) were seeded in 60-mm dishes and incubated for 48 h after treatment with 12 Gy irradiation. (A)Annexin V-FITC and PI (propidium iodide) staining was performed, followed by FACS analysis. (B) The percentage of apoptotic cells was counted (Figure 3A, areas 2 and 3). Similar results were obtained in three independent experiments. Errors bar represent the standard error of the mean (p < 0.05).

The apoptosis induced by 12 Gy irradiation was detected with Annexin V-FITC staining in cell lines EC109 and EC109/R. A significant difference was recognized between EC109 and EC109/R. As shown in figure 3B, about 1%–2% apoptosis was found in the control groups. In the radiation-treatment groups, the rate of apoptosis in EC109/R cells compared with EC109 cells was 6.81% ± 0.78% compared with 11.24% ± 1.21% at 48 h after treatment with 12 Gy irradiation (P < 0.05). Thus, the acquirement of radio-resistance was reflected in a reduced apoptotic rate.

### Cytotoxicity of cisplatin, 5-fluorouracil, doxorubicin, paclitaxel or etoposide on radio-resistant EC109/R cells

**Figure 4:**
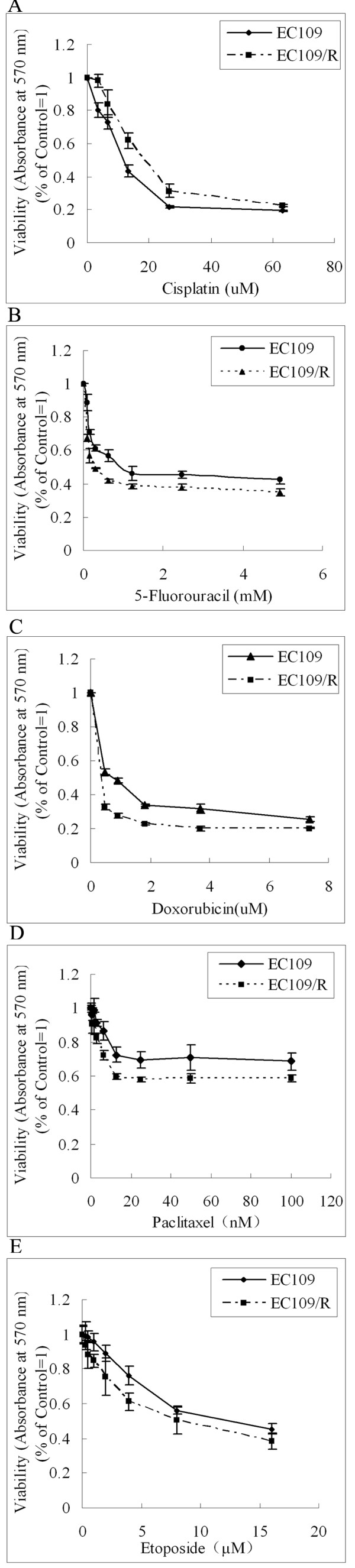
**Sensitivity of EC109 and EC109/R cells to cisplatin, 5-fluorouracil, doxorubicin, paclitaxel or etoposide**. EC109 or EC109/R Cells were exposed to various concentrations of cisplatin, 5-fluorouracil, doxorubicin, paclitaxel or etoposide for 48 h, and then the viability was calculated using MTT assay. Each point represents the mean ± SD of triplicate experiments (p < 0.05).

**Figure 5:**
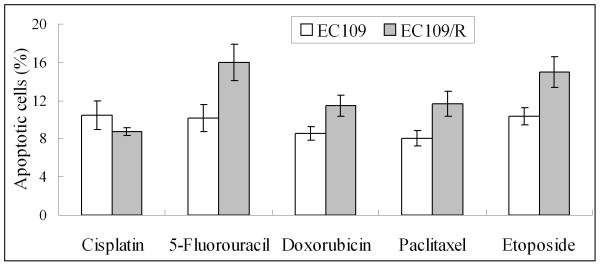
**Apoptotic changes in EC109 and EC109/R cells treated with different drugs**. EC109 and EC109/R cells treated with different drugs (10 μM cisplatin, 1000 μM 5-fluorouracil, 0.6 μM doxorubicin, 0.025 μM paclitaxel or 10 μM etoposide) for 48 hours were harvested by trypsinization and subjected to annexin V/propidium iodide apoptosis detection assay using a FACS flow cytometer. The percentage of apoptotic cells was counted (Figure 3A, areas 2 and 3). Similar results were obtained in three independent experiments. Errors bar represent the standard error of the mean (p < 0.05).

To examine if cellular resistance to ionizing radiation also causes cross-resistance to the chemotherapeutic agents, the effects of cisplatin, 5-fluorouracil, doxorubicin, paclitaxel and etoposide on the growth of EC109 or EC109/R cells were evaluated by determining cell viability using MTT assay. The dose-effect curves and IC_50_s to different treatment are shown in figure 4 and table 2. Compared with the parent cell line EC109, the IC_50 _value of EC109/R cells was 1.75-fold for cisplatin, 0.324-fold for 5-fluorouracil, 0.44-fold for doxorubicin, 0.64-fold for paclitaxel and 0.81-fold for etoposide. EC109/R cells were more sensitive than parental cells to 5-fluorouracil, doxorubicin, paclitaxel and etoposide. But the sensitivity of EC109/R to cisplatin decreased. In addition, the numbers of apoptotic cells were also determined by Annexin V staining followed by FACS analysis, which showed the same results (Figure 5). Radio-resistance increased sensitivity to chemotherapeutic drugs of 5-fluorouracil, doxorubicin, paclitaxel and etoposide significantly. But the radio-resistant subline was more resistant to cisplatin than the parent cell line EC109.

**Table 2:** Comparison of the cytotoxic effects of cisplatin, 5-fluorouracil, doxorubicin, paclitaxel or etoposideon on parental EC109 and EC109/R subline.

	IC_50_(uM)				
	
Cells	Cisplatin	5-Fluorouracil	Doxorubicin	Paclitaxel	Etoposide
EC109	10.99	923.8	0.67	0.0263	9.46
EC109/R	19.24	299	0.294	0.0169	7.69

Resistance index*	1.75	0.324	0.44	0.64	0.81

*Resistance index = (IC_50 _on EC109/R)/(IC_50 _on EC109)

## Discussion

Ionizing radiation (IR) is a potent agent in enhancing tumor control of locally advanced cancer and has been shown to improve disease-free and overall survival in several entities. Approximately 50%–70% of all cancer patients receive radiotherapy during their treatment. Advances in tumor imaging and physical targeting of IR and optimization of IR delivery schedules from single treatments to continuous irradiation have yielded significant improvements in patient outcome [16]. Nonetheless, many tumors are poorly controlled by radiotherapy alone.

Radio-resistance is an obstacle in cancer therapy and affects the curability of patients. Chronic exposure of cells to IR induces an adaptive response that results in enhanced tolerance to the subsequent cytotoxicity of IR [17]. In the present study, radio-resistant subline EC109/R was obtained by exposing the human ESCC cell line with 80 Gy of fractionated X-rays over an 8-month period. This results in a statistically significant decreased in the radiosensitivity of the exposed subline as messured by clonogenic assay. But the growth of EC109/R was similar to that of the parental cell line (Figure 2). One explanation for the increased radio-resistance might be an adaptive response to the selective pressure of repeated radiation. We observed that the radio-resistant subline maintained a radio-resistant phenotype for at least 2 months after cessation of fractionated irradiation in the absence of further treatment (data not shown).

Over the past several years, it has become increasingly evident that esophageal cancer is a disease that is potentially sensitive to chemotherapy. Recent data suggest that multimodal therapy is superior to single chemotherapy. Chemo-radiotherapy can be delivered as a definitive local therapy without surgery in the treatment of esophageal cancer [10]. The survival rates for chemo-radiation at 5 and 8 years were 32% and 22%, respectively. However, the optimal chemotherapy for advanced esophageal cancer remains unsettled, and there is no single standard regimen. The most common chemotherapy agents used in conjunction with radiation have been cisplatin, 5-fluorouracil and docetaxel. Several new chemotherapy agents are being tested in combination with radiation, but the best chemotherapy remains to be determined.

The fate of irradiated cells is believed to be controlled by the network of signaling elements that lead to different modes of cell death or survival. Many stress-responsive genes are inducible by IR [18,19]. These radiation-inducible genes are believed to have effects on the chemosensitivity of tumor cells [13,20]. To determine the correlation between radio-resistance and sensitivity to chemotherapeutic drugs in esophageal cancer cells, we then analyzed the chemosensitivity of EC109 and EC109/R cells with chemotherapeutic drugs cisplatin, 5-fluorouracil, doxorubicin, paclitaxel or etoposide. EC109/R, which survived 80 Gy irradiation, became more sensitive to different concentrations of 5-fluorouracil, doxorubicin, paclitaxel and etoposide, but maintained tolerance to cisplatin, as assessed by MTT assay (Figure 4). These findings suggest that cellular resistance to ionizing radiation have effects on the chemotherapeutic drug sensitivity in esophageal cancer cells.

Several genes associated with cellular sensitivity to anticancer drugs have been selected for esophageal cancer. They were B4GALT5 (UDP-Gal: βGlcNAc β1,4-galactosyltransferase, polypeptide 5 gene), UGCG (UDP-glucose ceramide glucosyltransferase gene), and XBP1 (X-box binding protein 1 gene) for 5-fluorouracil, NRCAM (neuronal cell adhesion molecule gene) for doxorubicin, ARFRP1 (ADP-ribosylation factor related protein 1 gene), IFITM1 (interferon induced transmembrane protein 1 gene), KIAA0685, and SIPA1L2 (signalinduced proliferation-associated 1 like 2 gene) for cisplatin [14]. Fractionated irradiation might induce cellular sensitivity related gene and protein expression in human tumor cell lines. The fact that drug sensitivity is determined by multiple genes required a better understanding of the intricate network of the selected genes in the expression levels.

Fractionated radiation treatment has also been reported to cause drug resistance in ovarian carcinoma cells [21] and ascites tumor cells [22]. It can induce functionally relevant multidrug resistance gene and protein expression in human tumor cell lines [13]. There are multiple factors that contribute to cisplatin resistance, but alterations of DNA repair processes have been known for some time to be important in mediating resistance [23,24]. The most important DNA repair pathways involved in the cisplatin response are nucleotide excision repair (NER) and mismatch repair (MMR). MSI, which results from disorder of the MMR system and loss of MLH1 protein, is frequently induced during cisplatin-based chemotherapy [25]. Data have shown that suppression of ERCC1 expression enhances or restores cisplatin sensitivity, and combination of p53 inactivation and MMR deficiency results in cisplatin resistance [26]. Moreover, enhancement of P-gp and MRP1 after irradiation was accompanied by a cisplatin-resistance phenomenon [13]. Our results are still preliminary, and further investigations are required to understand the mechanisms of the increased or decreased drug sensitivity in the radio-resistant cell line. As a next step, *in vivo *experiments would be necessary to confirm the relevance for radio-chemotherapy of cancer. A detailed understanding of the mechanisms of radiation-induced chemosensitivity may prove very helpful for choosing the sequence of radiotherapy and chemotherapy in esophageal cancer.

## Conclusion

Our study demonstrated a significant association between the cellular radio-resistance and the sensitivity of chemotherapeutic drugs in esophageal carcinoma cells. This result implied that doxorubicin, 5-fluorouracil, paclitaxel or etoposide will provide a more marked therapeutic effect for radio-resistant esophageal cancer. It will be important to confirm these findings and to take them into account in the development of new treatment sequence for ESCC.

## Competing interests

The authors declare that they have no competing interests.

## Authors' contributions

LX and LW carried out cell treatments and radiosensitivity assay; BS, XW and LL contributed to MTT cell viability assay and flow cytometry analysis. LX, XS and JY supervised experimental work and wrote the manuscript. All authors read and approved the final manuscript.

## References

[B1] Law S, Wong J (2007). The current management of esophageal cancer. Adv Surg.

[B2] Parkin DM, Bray F, Ferlay J, Pisani P (2005). Global cancer statistics, 2002. CA Cancer J Clin.

[B3] Seitz JF, Dahan L, Jacob J, Artru P, Maingon P, Bedenne L, Triboulet JP (2006). Esophagus cancer. Gastroenterol Clin Biol.

[B4] Enzinger PC, Mayer RJ (2003). Esophageal cancer. N Engl J Med.

[B5] Wright CD (2005). Esophageal cancer surgery in 2005. Minerva Chir.

[B6] Xiao ZF, Yang ZY, Liang J, Miao YJ, Wang M, Yin WB, Gu XZ, Zhang DC, Zhang RG, Wang LJ (2003). Value of radiotherapy after radical surgery for esophageal carcinoma: a report of 495 patients. Ann Thorac Surg.

[B7] Ku GY, Ilson DH (2007). Esophageal cancer: adjuvant therapy. Cancer J.

[B8] Brenner B, Ilson DH, Minsky BD (2004). Treatment of localized esophageal cancer. Semin Oncol.

[B9] Ku GY, Ilson DH (2008). Preoperative therapy in esophageal cancer. Clin Adv Hematol Oncol.

[B10] Liao Z, Cox JD, Komaki R (2007). Radiochemotherapy of esophageal cancer. J Thorac Oncol.

[B11] Ng T, Dipetrillo T, Purviance J, Safran H (2006). Multimodality treatment of esophageal cancer: a review of the current status and future directions. Curr Oncol Rep.

[B12] Carcaterrra M, Osti MF, De Sanctis V, Caruso C, Berardi F, Enrici RM (2005). Adjuvant radiotherapy and radiochemotherapy in the management of esophageal cancer: a review of the literature. Rays.

[B13] Bottke D, Koychev D, Busse A, Heufelder K, Wiegel T, Thiel E, Hinkelbein W, Keilholz U (2008). Fractionated irradiation can induce functionally relevant multidrug resistance gene and protein expression in human tumor cell lines. Radiat Res.

[B14] Shimokuni T, Tanimoto K, Hiyama K, Otani K, Ohtaki M, Hihara J, Yoshida K, Noguchi T, Kawahara K, Natsugoe S (2006). Chemosensitivity prediction in esophageal squamous cell carcinoma: novel marker genes and efficacy-prediction formulae using their expression data. Int J Oncol.

[B15] Song X, Liu X, Chi W, Liu Y, Wei L, Wang X, Yu J (2006). Hypoxia-induced resistance to cisplatin and doxorubicin in non-small cell lung cancer is inhibited by silencing of HIF-1alpha gene. Cancer Chemother Pharmacol.

[B16] Suit H (2002). The Gray Lecture 2001: coming technical advances in radiation oncology. Int J Radiat Oncol Biol Phys.

[B17] Ogawa K, Utsunomiya T, Mimori K, Tanaka F, Haraguchi N, Inoue H, Murayama S, Mori M (2006). Differential gene expression profiles of radioresistant pancreatic cancer cell lines established by fractionated irradiation. Int J Oncol.

[B18] Gupta S, Ahmed MM (2004). A global perspective of radiation-induced signal transduction pathways in cancer therapeutics. Indian J Exp Biol.

[B19] Ahmed KM, Dong S, Fan M, Li JJ (2006). Nuclear factor-kappaB p65 inhibits mitogen-activated protein kinase signaling pathway in radioresistant breast cancer cells. Mol Cancer Res.

[B20] Ryu JS, Um JH, Kang CD, Bae JH, Kim DU, Lee YJ, Kim DW, Chung BS, Kim SH (2004). Fractionated irradiation leads to restoration of drug sensitivity in MDR cells that correlates with down-regulation of P-gp and DNA-dependent protein kinase activity. Radiat Res.

[B21] Hill BT, Moran E, Etievant C, Perrin D, Masterson A, Larkin A, Whelan RD (2000). Low-dose twice-daily fractionated X-irradiation of ovarian tumor cells in vitro generates drug-resistant cells overexpressing two multidrug resistance-associated proteins, P-glycoprotein and MRP1. Anticancer Drugs.

[B22] Nielsen D, Maare C, Eriksen J, Litman T, Skovsgaard T (2001). Expression of P-glycoprotein and multidrug resistance associated protein in Ehrlich ascites tumor cells after fractionated irradiation. Int J Radiat Oncol Biol Phys.

[B23] Martin LP, Hamilton TC, Schilder RJ (2008). Platinum resistance: the role of DNA repair pathways. Clin Cancer Res.

[B24] Borst P, Rottenberg S, Jonkers J (2008). How do real tumors become resistant to cisplatin?. Cell Cycle.

[B25] Watanabe Y, Koi M, Hemmi H, Hoshai H, Noda K (2001). A change in microsatellite instability caused by cisplatin-based chemotherapy of ovarian cancer. Br J Cancer.

[B26] Lin X, Howell SB (2006). DNA mismatch repair and p53 function are major determinants of the rate of development of cisplatin resistance. Mol Cancer Ther.

